# Associations between the Cervical Vertebral Column and Craniofacial Morphology

**DOI:** 10.1155/2010/295728

**Published:** 2010-06-15

**Authors:** L. Sonnesen

**Affiliations:** Department of Orthodontics, Institute of Odontology, Faculty of Health Sciences, University of Copenhagen, 2200 Copenhagen, Denmark

## Abstract

*Aim*. To summarize recent studies on morphological deviations of the cervical vertebral column and associations with craniofacial morphology and head posture in nonsyndromic patients and in patients with obstructive sleep apnoea (OSA). *Design*. In these recent studies, visual assessment of the cervical vertebral column and cephalometric analysis of the craniofacial skeleton were performed on profile radiographs of subjects with neutral occlusion, patients with severe skeletal malocclusions and patients with OSA. Material from human triploid foetuses and mouse embryos was analysed histologically. *Results*. Recent studies have documented associations between fusion of the cervical vertebral column and craniofacial morphology, including head posture in patients with severe skeletal malocclusions. Histological studies on prenatal material supported these findings. *Conclusion*. It is suggested that fusion of the cervical vertebral column is associated with development and function of the craniofacial morphology. This finding is expected to have importance for diagnostics and elucidation of aetiology and thereby for optimal treatment.

## 1. Introduction

Cephalometric analyses of the cervical vertebral column area have previously been performed on profile radiographs. It was found that the horizontal and vertical dimensions of the first cervical vertebra (C1), atlas, were associated with head posture, cranial base angulation, and mandibular shape and growth [1–4]. Also, posture of the head and neck was associated with factors such as craniofacial morphology including the cranial base [5–11], upper airway space [9, 12, 13], to some extent occlusion [14, 15], and temporomandibular disorders [16–21]. Many cross-sectional studies agree on a relationship between extended head posture and craniofacial structures [1, 5–10]. In subjects with extended head posture, increased anterior facial height, reduced sagittal jaw dimensions, and a steeper inclination of the mandible were generally observed. When the head was bent in relation to the cervical column, a shorter anterior facial height, larger sagittal jaw dimensions, and a less steep inclination of the mandible were observed. Some longitudinal studies likewise demonstrated that growth changes in head posture were related to corresponding changes in the growth pattern of the facial skeleton [22, 23]. When the head was extended, a reduced forward rotation of the mandible was observed. 

Cephalometric studies of the cervical vertebral column area have also been performed on patients with obstructive sleep apnoea. Most of these studies agree that patients with obstructive sleep apnoea have an extended head posture [12, 24–31]. 

Until recently, deviations of the cervical vertebral column have only been described in relation to craniofacial syndromes and cleft lip and palate. Craniosynostosis syndromes, for example, Pfeiffer's, Crouzon's, and Apert's syndromes, showed deviations such as fusion anomalies [32–36]. Furthermore, deviations of the cervical column morphology were seen in Saethre-Chotzen, Klippel-Feil, Turner, and Down syndromes [37–41]. Also, malformations of the upper cervical vertebrae have been closely investigated in patients with cleft lip and/or palate [42–47].

Accordingly, associations have been reported between head posture and craniofacial morphology, between head posture and OSA, and between morphological deviations of the cervical vertebral column and craniofacial syndromes [32–36, 42–47] and cleft lip and/or palate. The aim of the present study was to summarize recent studies on morphological deviations of the cervical vertebral column and the associations with the craniofacial skeleton and head posture in nonsyndromic patients and in patients with obstructive sleep apnoea and to elucidate the aetiology behind the associations as well as clinical implications of the results.

## 2. Definition of Morphological Deviations of the Cervical Vertebral Column

The cervical vertebral column morphology of the upper five cervical vertebrae (C1–C5) on a lateral skull radiograph is divided into two main categories: “Fusion Anomalies” and “Posterior Arch Deficiency” according to Sandham, 1986 [43].

**Figure 1:**
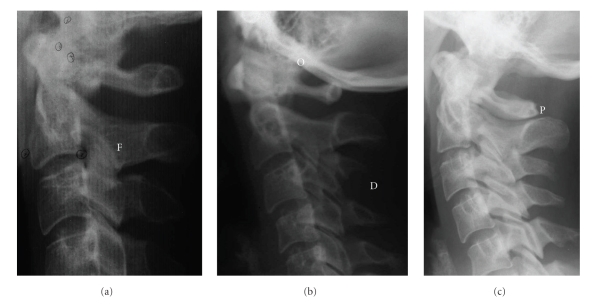
Illustrations of morphological deviations of the upper cervical vertebral column. F: Fusion is defined as fusion of one unit with another at the articulation facets, neural arch, or transverse processes. O: Occipitalization is defined as assimilation, either partially or completely, of the atlas (C1) with the occipital bone. P: Partial cleft is defined as failure to fuse of the posterior part of the neural arch. D: Dehiscence is defined as failure to develop of part of a vertebral unit.

Fusion anomalies are fusion, block fusion, and occipitalization [43]. Fusion is defined as fusion of one unit with another at the articulation facets, neural arch, or transverse processes (Figure 1). Occipitalization is defined as assimilation, either partially or completely, of the atlas (C1) with the occipital bone (Figure 1). The definition of block fusion has been modified according to Sonnesen and Kjær [48] and defined as fusion of more than two units at the vertebral bodies, articulation facets, neural arch, or transverse processes. 

Posterior arch deficiency consists of partial cleft and dehiscence according to Sandham, 1986 [43]. Partial cleft is defined as failure to fuse of the posterior part of the neural arch (Figure 1). Dehiscence is defined as failure to develop of part of a vertebral unit.

## 3. Association between Cervical Vertebral Column Morphology and Craniofacial Morphology

Sonnesen et al. have recently described morphological deviations of the cervical vertebral column in healthy subjects with neutral occlusion and normal craniofacial morphology [49, 50] and in patients with severe skeletal malocclusion traits such as skeletal deep bite, skeletal open bite, skeletal maxillary overjet, and skeletal mandibular overjet [48, 51–53]. It was found that morphological deviations of the cervical vertebral column such as fusion occurred significantly more often in patients with severe skeletal malocclusion traits when compared to controls. Fusion in the control groups occurred in 14–21 percent (*N* = 3–8), and fusions were always seen between the second and third cervical vertebrae [49, 50]. Fusion of the cervical vertebral column in the severe skeletal malocclusion groups occurred in 41–61 percent (*N* = 17–35). In the deep bite group, the open bite group, and in the horizontal maxillary overjet group, fusions were always seen between the second and third cervical vertebrae. The same pattern was seen in the control group [51–53]. The pattern of fusion in the mandibular overjet group differed from that of the control group as not only fusion occurred between the second and third cervical vertebrae but also block fusion between the second, third, and fourth cervical vertebrae [48]. In patients with condylar hypoplasia the pattern of fusion also differed as fusion between the third and fourth cervical vertebrae and occipitalization occurred [49]. These findings indicate an association between fusion of the cervical vertebral column and severe skeletal malocclusion.

A series of recent studies have focussed on the association between morphological deviations of the cervical vertebral column and the craniofacial morphology in adult patients with severe skeletal malocclusion traits [48, 51–53]. These studies revealed that fusion of the cervical vertebral column had the closest association with craniofacial morphology. Significant associations between fusion and a large cranial base angle, between fusion and retrognathia of the jaws, and between fusion and inclination of the jaws were found in patients with severe skeletal malocclusions. The same pattern of craniofacial morphology was found in monozygotic twins when twins with fusion of the cervical vertebral column were compared with twins without fusion [50]. These findings indicate an association between fusion of the cervical vertebral column and the craniofacial morphology including the cranial base.

## 4. Association between Cervical Vertebral Column Morphology and Head Posture

An association between posture of the head and neck and the cervical vertebral column morphology has recently been demonstrated by Sonnesen et al. [49]. In individuals with neutral occlusion and normal craniofacial morphology, the cervical lordosis was significantly more curved and the inclination of the upper cervical column was more backwards in individuals with fusion than in individuals without fusion [49]. These findings indicate an association between fusion of the cervical column and posture of the neck.

## 5. Association between Cervical Vertebral Column Morphology and Sleep Apnoea

A study by Sonnesen et al. on cervical vertebral column morphology in patients with OSA [54] found a 46-percent (*N* = 42) prevalence of fusion anomalies in the cervical vertebral column. The deviations occurred significantly more often in patients with OSA and at a lower level in the cervical vertebral column compared to controls [54]. Fusion anomalies occurred as fusions either between the second and third vertebrae, between the third and fourth vertebrae, or between the fourth and fifth cervical vertebrae. Block fusions occurred as fusions either between the second, third, or fourth vertebrae, between the second, third, fourth, and fifth vertebrae, or between the third, fourth, and fifth vertebrae. Occipitalization occurred in combination with fusions, block fusions or as a single deviation [54]. 

The results of the studies on subjects with neutral occlusion and patients with severe skeletal malocclusions suggest that fusion of the cervical vertebral column is associated with occlusion, craniofacial morphology, and head posture. Furthermore, a different morphological pattern of the cervical vertebral column was found in patients with OSA.

## 6. Discussion

Associations between craniofacial morphology and head posture and between head posture and obstruction of the upper airways seen in patients with OSA have previously been demonstrated. Recent findings have established associations between craniofacial morphology including the cranial base and fusion of the cervical vertebral column, between head posture and fusion of the cervical vertebral column and between patients with OSA and fusion of the cervical vertebral column. 

**Figure 2:**
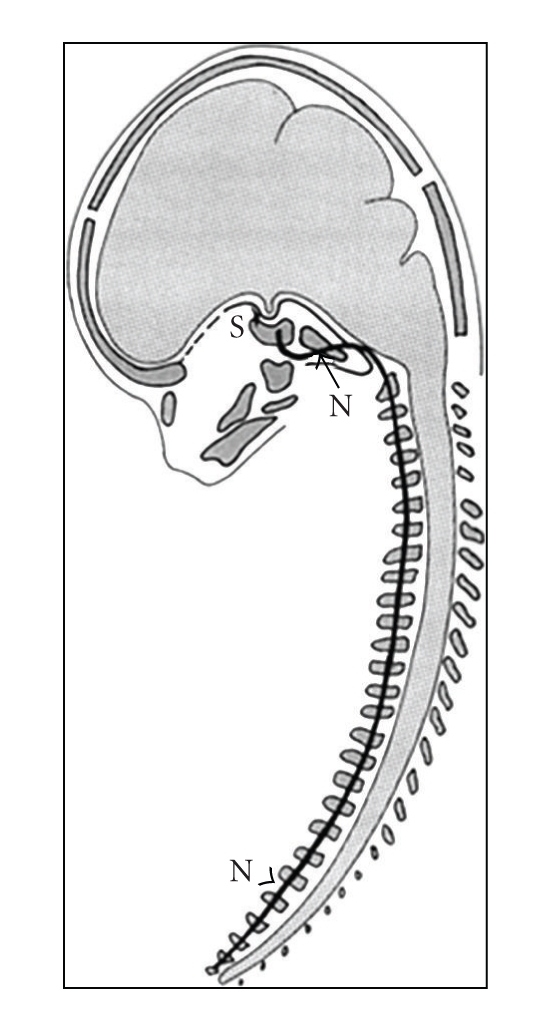
Illustration of the extension of the notochord (N). The black line indicates the caudocranial extension of the notochord through the vertebral bodies and the posterior part of the cranial base.

An explanation for the association between fusion of the cervical column and a large cranial base angle could be found in the early embryogenesis. The notochord develops in the human germ disc and determines the development of the cervical vertebrae, especially the vertebral bodies, and also the basilar part of the occipital bone in the cranial base [55–59] (Figure 2). The para-axial mesoderm forming the vertebral arches and remaining parts of the occipital bone are also formed from notochordal inductions. Therefore, a deviation in the development of the notochord may influence the surrounding bone tissue in the vertebral column as well as in the posterior part of the cranial base. It can be observed on postnatal profile radiographs that the bone tissue formed around the notochord is the vertebral bodies and the basilar part of the occipital bone. The shared origin of the vertebral column and the posterior part of the cranial base supports the new hypothesis that associations between the cervical vertebral column and the cranial base exist [60, 61]. 

The association between fusion of the cervical column and the craniofacial morphology could also be explained by the early embryogenesis. It is known that the neural crest cells migrate to the craniofacial area before the notochord is surrounded by bone tissue and disappears [55, 57–59, 61, 62]. The jaws, including the condylar cartilage, develop from ectomesenchymal tissue derived by the neural crest. In the first branchial arch the neural crest cells migrate from the neural crest towards the mandible, followed by the cells to the maxilla and lastly by the cells to the nasofrontal region [57]. Therefore, it is understandable that a deviation in the amount or timing of migrating maxillary and mandibular cells may influence the craniofacial development [53]. The precise signalling from the notochord to the neural crest followed by bilateral cell migration to the craniofacial area is still unknown. Signalling during early embryogenesis between the notochord, para-axial mesoderm, the neural tube, and the neural crest may explain the association between the cervical vertebral column, cranial base, and craniofacial development [61].

The study on patients with obstructive sleep apnoea found that the prevalence of fusion anomalies of the cervical vertebral column occurred significantly more often in patients with OSA and at a lower level in the cervical vertebral column compared to controls [54]. These deviations in prevalence and pattern of the cervical vertebral column may prove a factor in the pathogenetic background of sleep apnoea and thereby contribute to the diagnosis and treatment of patients with OSA [54].

## 7. Conclusions

Recent findings have demonstrated associations between craniofacial morphology including the cranial base and fusion of the cervical vertebral column, between head posture and fusion of the cervical vertebral column and between patients with OSA and fusion of the cervical vertebral column. Accordingly, the results of these studies suggest that fusion of the cervical vertebral column is associated with the development and function of the craniofacial morphology. The morphological pattern of the upper cervical vertebrae is expected to be of importance for diagnostics and the elucidation of aetiology and thereby for the optimal treatment of patients with severe skeletal malocclusions and patients with OSA. 

These associations between the cervical vertebral column, the cranial base, and the craniofacial morphology suggest a new hypothesis: signalling during early embryogenesis between the notochord, para-axial mesoderm, the neural tube, and the neural crest explains these associations between the cervical vertebral column, the cranial base, and the craniofacial skeleton. 

It is suggested that dentists look at the cervical vertebral column area and register any deviations in the cervical vertebral column morphology and head posture. These registrations may prove useful when considering diagnosis and evaluating aetiology, especially in patients with severe skeletal malocclusions and OSA.
